# Intelligence

**Published:** 1808-04

**Authors:** 


### INTELLIGENCE.

### Medical Society of London.

This Society held its anniversary meeting on Tuesday, March 8. After the election of officers for the ensuing year, Mr. Good, a distinguished member of this learned body, delivered an extempore oration, the subject of which was, A Survey of the Theory of the general structure and physiology of plants compared with those of animals; the process by which vegetable matter is converted into animal matter, so as to become the basis of animal nutriment and support; and the mode by which animal matter is afterwards reconverted into vegetable, so as to complete the circle of action, and to restore to plants the nutritive benefit which has been antecedently derived from them. —— In this interesting discourse, Mr. Good took a comprehensive view of the wisdom and goodness of the Creator, which pervades the universe, and is manifested throughout the various and innumerable links of that chain by which the varieties of organized beings are bound together, and rendered mutually dependent on each other. ——At the unanimous request of of the Society, Mr. Good has consented to publish this Oration in as exact a form as his recollection will serve him. ——The concourse of Members, as well as of Visitors, who were present on the occasion, exceeded that of any former anniversary.

---

Mr. Joseph Jewel has invented a new process of producing calomel that shall always be in a state of an impalpable powder. This is effected by a particular manipulation in the last sublimation of the calomel, which he describes as follows:—"I take calomel or mercurius dulcis, broken into small pieces, and put it into an earthern crucible of the form of a long bowl, so as to fill about one half of it. I place the crucible on its side in a furnace provided with an opening, through which the mouth of the crucible projects about an inch. I then join to the mouth of the crucible an earthen ware receiver, having an opening at its side, to receive the open end of the crucible. The receiver is about half filled with water. I lute the joint with a mixture of sand and pipe clay. The receiver has a cover, that has a side continued upwards for containing water, with a chimney or tube in it to allow the escape of steam from the water below. I then apply a fire round the crucible, sufficient to raise calomel in vapours, and force it through the mouth of the crucible into the receiver; where, by the water, while cold, or assisted by the steam when it becomes hot, it is instantly condensed into an impalpable powder, possessing all the qualities of calomel in its most perfect state. The calomel, when thus prepared, is purer, whiter, and more attenuated than that obtained by grinding. It is proper to wash the product over with water, before it is dried, to rid it of the coarser particles which may form about the mouth of the crucible."

---

Mr. Joseph Hume has published some observations on the use of sulphur as a vermifuge, and the proper way of applying it to vegetables. The method is extremely simple, for nothing more is required than to sprinkle sublimed sulphur, or what is commonly called flowers of brimstone, over the leaves of the tree or plant wherever the effects of worms, or insects, prevail. The sulphur may be tied up in a piece of muslin or linen, and with this, the leaves and young shoots should be dusted, or it may be thrown on by means of a puff, or a dredging box. This application is found not only to be effectual in destroying the whole tribe of worms and other insects which prey upon vegetables, but it is likewise ascertained to be congenial to the trees and plants on which it is sprinkled. Peach-trees, in particular, are remarkably improved by it.

---

Mr. Donevan has announced some particulars of an extraordinary nature, respecting one of the mountains of Wales, which he endeavours to demonstrate to have been, at some remote period, a volcano of immense magnitude. This is Cader Idris, in Merionethshire, which in size, is not exceeded by any mountain in in the principality, except Snowdon. The general aspect of the crater is exactly that of Mount Vesuvius, only one of its sides is broken down, so that the abyss of this funnel-shaped mountain is more completely disclosed than in the latter. It is this side of Cader Idris that affords the most illustrative examples of porous stones, which form immense beds on the declivities, only a few inches below the surface of the earth. Many of these porous stones exhibit evident marks of strong ignition and vitrification; some are reduced to the state of slags, while others have all the cellular appearance and lightness of pumice. The summit of the mountain is covered with an immense wreck of stones, supposed to have been ejected from the crater at the time of the explosion. Myriads of these stones have borne a regular crystallized form; their usual length is, from three to six and ten feet; some measure even fifteen or twenty, and one in particular, which Mr. Donevan observed, was twenty-two feet three inches long. The substance of these crystals is of the basalt kind, being the porphyry slate, or clinkstone porphyry of Jamieson.

---

Mr John Gough, of Middleshaw, has published some interesting observations on domesticated dormice, which strongly tend to invalidate the received theory of torpidity. The account which he gives of his experiments is as follows: "Having procured two dormice, in January, 1792, which were caught in the woods but a few days before they came into my hands, I confined them in a cage, furnished with a thermometer, and placed in a chamber where no fire was kept. They were supplied regularly with water and food, consisting of hazel-nuts and biscuits. The weather in February being warm for the season at the beginning and end of the month, and frosty from the 16th to the 28th, I had an opportunity to observe that whenever the thermometer, which was attached to the cage, fell to 42°, the dormice became inactive, and remained apparently insensible as long as the heat of that part of the chamber did not exceed the above-mentioned temperature; but whenever the mercury reached 47° they became very susceptible of external impression, and awaked in the evenings, when they repaired to their stock of provisions, of which they consumed not a little. The same dry food being injudiciously continued through the summer, they grew sickly and died, so that I had not a second opportunity to attend to the economy of this couple during the cold season. About the middle of April, 1793, I obtained a third dormouse. Experience taught me to manage this in a manner more congenial to its constitution. In addition to the nuts and biscuits, it was constantly supplied with green hazel buds or raisins in spring; with ripe fruits in summer, and with apples and raisins in winter. This generous diet not only preserved the creature in health and high condition, but appeared to fortify it against the benumbing effects of cold, which it supported the following winter much better than the other couple had done; for it never slept more more than forty-eight hours, and that but seldom, without visiting the cup which contained its provisions. I now began to suspect the torpidity of the dormouse, in a wild state, to be nothing but a custom imposed by necessity on a constitution which nature has intended to retain life during the cold season of winter, with but little food and an imperfect degreee of respiration, as well as a languid, or, perhaps, partial action of the sanguiferous system. The uncommonly severe weather which ushered in the year 1795, confirmed this opinion apparently beyond dispute; for notwithstanding the hard frost, it braved the cold with wonderful indifference. It awaked every evening, when it consumed in the course of the night a quantity of food amounting to one hundred, or one hundred and twenty grains, and frequently gnawed the ice which covered the water in the cage. It even undertook, in the coldest part of January, to repair its nest, which happened to receive an injury, and accomplished the task in one night."

---

Mr. ROBERTON, of Edinburgh, Author of The Practical Treatise on Gleet, Leucorrhœa, and Obstinate Sores, is preparing for the press, a work entitled The Diseases of Edinburgh; in which the sources of the permanent or regularly recurring diseases of Edinburgh are pointed out, and the entire removal of these sources, as well as the method of cure of the diseases they induce, are explained; preceded by a copious introduction, describing the general influence of local circumstances in the generation of disease, and detailing fully a general plan of Medical Police. This work will be divided into four parts, viz. 1. Of the general local Causes of Disease. 2. Of the Police to obviate them. 3. Of the local Causes of Disease in Edinburgh. 4. Of a Plan of Edinburgh Medical Police.

---

Mr. WADLEY, Surgeon, at Wootton Basset, has in the press A Letter on the Subject of Vaccination, addressed to those classes of the community, whose example may influence the inferior orders. It will be published early in April.

